# When intuition falters: repeated testing accuracy during an epidemic

**DOI:** 10.1007/s10654-021-00786-w

**Published:** 2021-07-28

**Authors:** James A. Hay, Joel Hellewell, Xueting Qiu

**Affiliations:** 1 Center for Communicable Disease Dynamics, Department of Epidemiology, Harvard T.H. Chan School of Public Health, Boston, MA USA; 2 Centre for Mathematical Modelling of Infectious Diseases, London School of Hygiene & Tropical Medicine, London, UK

**Keywords:** SARS-CoV-2, COVID-19, Lateral flow test, Rapid antigen test, Mass testing, Surveillance testing

## Abstract

Widespread, repeated testing using rapid antigen tests to proactively detect asymptomatic SARS-CoV-2 infections has been a promising yet controversial topic during the COVID-19 pandemic. Concerns have been raised over whether currently authorized lateral flow tests are sufficiently sensitive and specific to detect enough infections to impact transmission whilst minimizing unnecessary isolation of false positives. These concerns have often been illustrated using simple, textbook calculations of positivity rates and positive predictive value assuming fixed values for sensitivity, specificity and prevalence. However, we argue that evaluating repeated testing strategies requires the consideration of three additional factors: new infections continue to arise depending on the incidence rate, isolating positive individuals reduces prevalence in the tested population, and each infected individual is tested multiple times during their infection course. We provide a simple mathematical model with an online interface to illustrate how these three factors impact test positivity rates and the number of isolating individuals over time. These results highlight the potential pitfalls of using inappropriate textbook-style calculations to evaluate statistics arising from repeated testing strategies during an epidemic.

**Supplementary Information:**

The online version contains supplementary material available at 10.1007/s10654-021-00786-w.

From 8 March 2021, students in English secondary schools began routine asymptomatic testing for SARS-CoV-2 infection using rapid lateral flow tests (LFTs). Students were tested at school three times during the first two weeks after their return to the classroom and then given two tests per week to use at home. The strategy aimed to identify and prompt the isolation of infected students to keep SARS-CoV-2 transmission within schools minimal.

Two primary concerns were raised over widespread LFT surveillance testing in schools. First, the observed test positivity rate from LFT testing was lower than SARS-CoV-2 prevalence estimates from population-wide surveys [1, 2]. This raised the question of whether many infections were being missed due to inadequate LFT sensitivity. Second, as the prevalence of SARS-CoV-2 in school aged children was low and declining, it was argued that a high number of false positives results could lead to considerable unnecessary isolation for students and their class bubbles. These concerns have largely been illustrated using simple, textbook calculations that assume fixed numbers for test sensitivity, specificity and prevalence measured at a single point in time.

We argue that interpreting statistics from repeated surveillance testing requires the consideration of three additional factors: (1) infection incidence changes over time, dictating not only prevalence but also the number of new infections to be detected, (2) isolating detected infections decreases prevalence in the tested population with each subsequent testing round, and (3) test sensitivity is not a static value but varies over the course of an individual’s infection. Using a simple model of repeated LFT testing, we illustrate how these factors influence the test positivity rate and positive predictive value (PPV) of a given testing strategy, thereby dictating the number of incorrectly isolating individuals over time. Although the model is broadly based on testing in English secondary schools, these considerations are relevant for interpreting statistics from repeated testing strategies in general. The model is available to test under alternative assumptions in an accompanying online tool.

The first observation from the model is that positivity rates under regular testing are lower than prevalence observed in random cross-sectional LFT/qPCR surveys. Although routine LFT screening initially returns the same percentage positive as a random cross-sectional survey, the number of true positives left to be detected is reduced with each testing round as true positives are detected, isolated and no longer retested. This effect may be counteracted by the incidence of new infections, but with a sufficiently high testing frequency the pool of true positives will be depleted faster than it can be replenished. As a result, the percentage of positive tests under a regular screening strategy sits between incidence and prevalence (Fig. 1a)—a low observed test positivity compared to cross-sectional surveillance is therefore expected under regular screening and should not be compared to prevalence estimates to evaluate the success of the strategy.

**Fig. 1:**
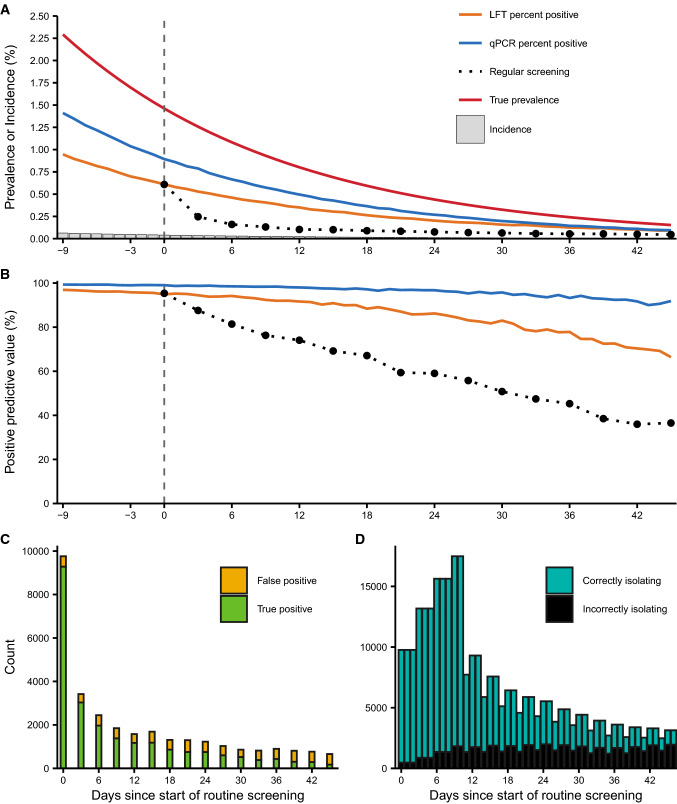
Test positivity and positive predictive value decline alongside prevalence, but the number of false positives and subsequent isolations are stable. Simulation uses a population of 1.5 million individuals to match the number of secondary school pupils tested in their first week of return to school, with prevalence, test characteristics and test frequency chosen to broadly reflect the situation in England 7. True prevalence, defined as the prevalence of all individuals within 21 days of infection, ranged from 2.29% on the first day of the strategy to 0.153% on the last day. LFT specificity is set to 99.97% and qPCR specificity to 99.99%. Full simulation assumptions and parameters are shown in the electronic supplementary material. The repeated screening strategy is initiated when true prevalence is at approximately 1.5% (vertical dashed lines). (A) Percentage of tests returning positive under random cross-sectional testing with LFT or qPCR and no isolation of positives or testing all individuals by LFT every three days with isolation of positives compared to true prevalence and incidence. Note that daily incidence ranges from 6.33 to 0.405 infections per 10,000 people (Supplementary Information Fig. S4). Black dots show the observed test positivity from the 3-day LFT screening tests performed on that day. (B) Positive predictive value (PPV) of random cross-sectional testing with LFT or qPCR, or 3-day LFT screening with removal of positives. PPV ranged from 95.3% to 36.5% for the 3-day LFT screening strategy. (C) Number of positives detected on each day of the 3-day LFT screening strategy. (D) Number of individuals in isolation over time under the 3-day LFT screening strategy, stratified by whether they were detected as a true or false positive

The second observation is that the removal of true infections from the tested population affects the proportion, but not absolute number, of false positive test results. The probability that a positive LFT represents a true infection is given by the PPV, which depends on the test specificity and prevalence of SARS-CoV-2. Although both LFTs and qPCR are highly specific (LFT specificity has been estimated at 99.5% or higher [3, 4]), PPV will be low during periods of very low prevalence. Because prevalence in the test population decreases with each testing round as true positives are detected and isolated, the PPV of the regular testing program declines over time, on top of any effect from changes in overall prevalence (Fig. 1b). Conversely, the negative predictive value increases with each testing round (Supplementary Information Fig. S1).

It is important to distinguish the PPV from the false positive rate, which is independent of prevalence and given by the number of false positives divided by the number of tests performed on truly uninfected individuals [5]. While the PPV of the testing strategy may become low, the absolute number of false positives is constant and remains small in each testing round. This means that a relatively small number of individuals are incorrectly isolating at a given time (Fig. 1c, d), unless a large number of contacts, such as pupils from the same class, are also quarantined following detection of a single positive.

The final observation is that very few infections go completely undetected when individuals are routinely tested every three days, as at least one test day will likely coincide with a period of high viral load (Supplementary Information Fig. S2). LFT sensitivity has been reported as a single value at 50.1% in the context of school testing, reflecting the overall positive percentage agreement (PPA) with qPCR for infected individuals tested on an unknown day post infection [3]. However, test sensitivity is not a single static value, but depends on the quantity of viral material within the host, which increases and then decreases over the course of an infection (Supplementary Information Fig. S3). When individuals are tested prospectively, infections that are missed due to low viral loads on the first test day are likely to have higher viral loads and corresponding increased sensitivity at the next test: the sensitivity of the overall strategy to detect an infection in at least one test is very high, even if the observed sensitivity from trials involving testing individuals once is low.

Overall, these observations demonstrate the challenges of interpreting positivity rates when repeatedly testing and isolating individuals during periods of declining SARS-CoV-2 prevalence. Simple calculations assuming fixed values for test characteristics and prevalence are useful for illustrative purposes but are insufficient to interpret statistics emerging from repeated testing programs during an epidemic. A related topic not discussed here is the choice of test characteristics tailored to different public health purposes [6]. Here, we defined a true positive as any individual within 21 days of infection, but we note that more stringent definitions including only infectious or high viral load individuals will also impact the interpretation of LFT and qPCR positivity rates. Although the ability of sustained mass screening strategies, as carried out in English schools, to detect and isolate infected individuals is very high, the costs, benefits and risk of false negatives arising from confirmatory and test-to-release antigen or molecular testing will require ongoing evaluation.

## Supplementary Information

Below is the link to the electronic supplementary material.

Supplementary file1 (DOCX 300 kb)

## Data Availability

Not applicable. This is a simulation study.

## Code availability

An online tool to run the model under alternative assumptions and parameter values is available at https://jamesahay.shinyapps.io/screening_accuracy/. All code available to reproduce the analyses are available at https://github.com/jameshay218/screening_accuracy.
