# Supplementary material for: When intuition falters: repeated testing accuracy during an epidemic
Source: Eur J Epidemiol. 2021 Jul 28;36(7):749–52. doi: 10.1007/s10654-021-00786-w (PMC8318052; doi:10.1007/s10654-021-00786-w)
Supplement: Supplementary file 1 — Supplementary file1 (DOCX 300 kb) [file 10654_2021_786_MOESM1_ESM.docx]

**When intuition falters: repeated testing accuracy during an epidemic**

**Supplementary Information**

James A. Hay*^1^, Joel Hellewell^2^, Xueting Qiu^1^

***[*jhay@hsph.harvard.edu*](mailto:jhay@hsph.harvard.edu)

1. Center for Communicable Disease Dynamics, Department of Epidemiology, Harvard University T H Chan School of Public Health, Boston, Massachusetts, USA.

2. Centre for Mathematical Modelling of Infectious Diseases, London School of Hygiene & Tropical Medicine, UK


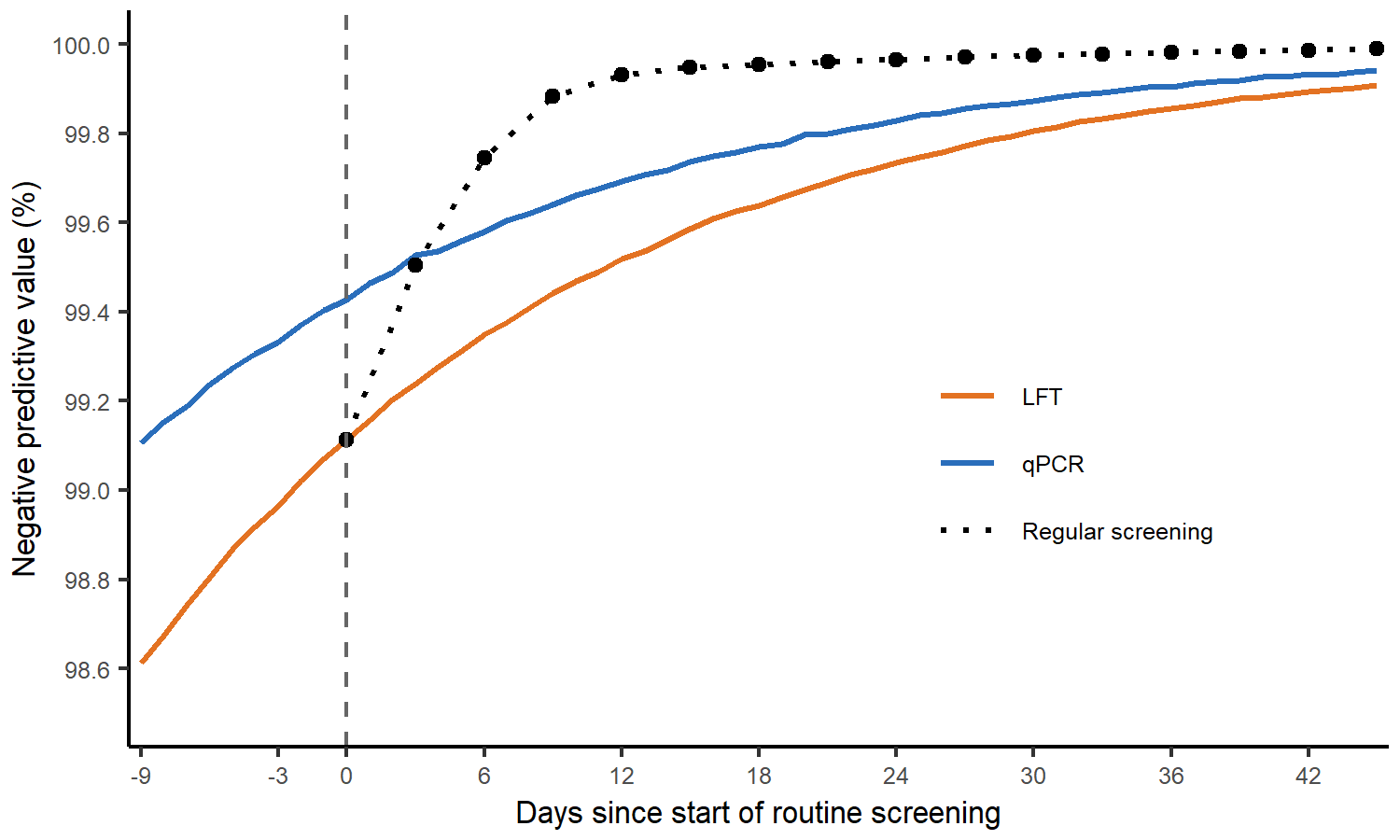


**Supplementary Figure 1.** Negative predictive value (NPV) of daily testing of all individuals with qPCR or LFT versus LFT testing and isolating positive individuals every three days. Dashed vertical line shows the time when the regular screening strategy is initiated. NPV ranged from 99.1% to 100% for the 3-day LFT screening strategy.

**
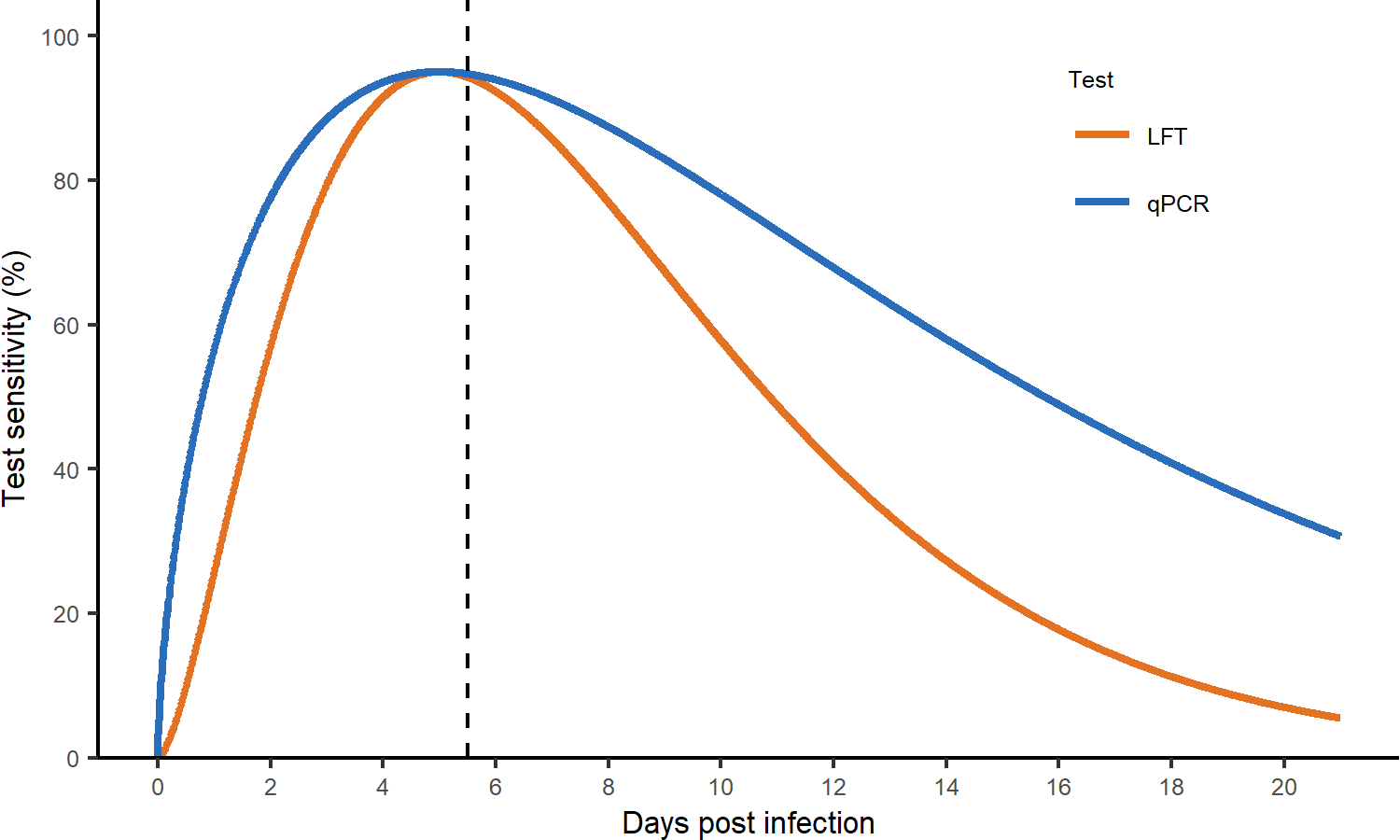
**

**Supplementary Figure 2.** Assumed test sensitivity over time post infection. That is, the probability of returning a positive test result given a sample is taken on a given day after infection. The dashed vertical line shows day 5.5 post infection as a benchmark for the typical time of symptom onset. Under these curves, the mean LFT and qPCR sensitivity over the 21-day period are 44% and 63% respectively. The mean sensitivities over days 2–12 post infection are 71% and 83% for LFT and qPCR respectively.


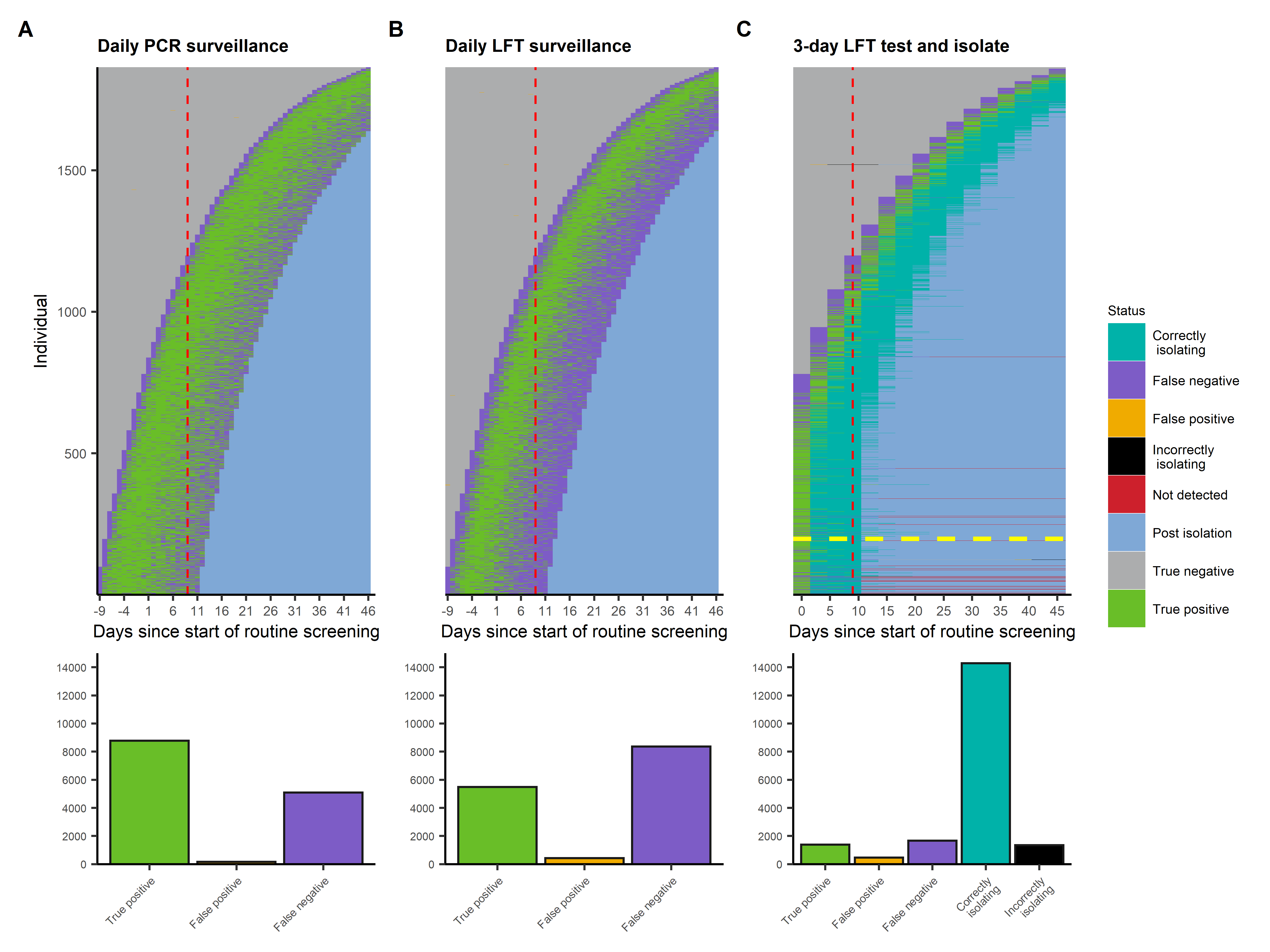


**Supplementary Figure 3.** (Top) Individual-level test results under complete surveillance with no isolation using (A) qPCR, (B) LFT or (C) testing all individuals every three days with an LFT followed by 10-day isolation of positives. Each row shows one individual, ordered by infection date. Plot is subsampled to show 10% of the full population. Each row is an individual, coloured to show their infection status on each day of the simulation. Note that only individuals who are infected within the focal period are plotted, and all uninfected individuals would be plotted as predominantly grey bars with infrequent false positives. The vertical red dashed line shows a single observation time for the bar charts shown in the bottom row. In (C), the horizontal yellow line gives a boundary of old versus new infections: individuals below this line were infected in the nine days prior to the start of the regular screening strategy, and thus represent older infections. Most infections who are not detected before they recover occurred prior to the start of routine screening; there are very few individuals infected after the start of the screening strategy who escaped detection (see *Supplementary Material 1: Methods* for further results).

**
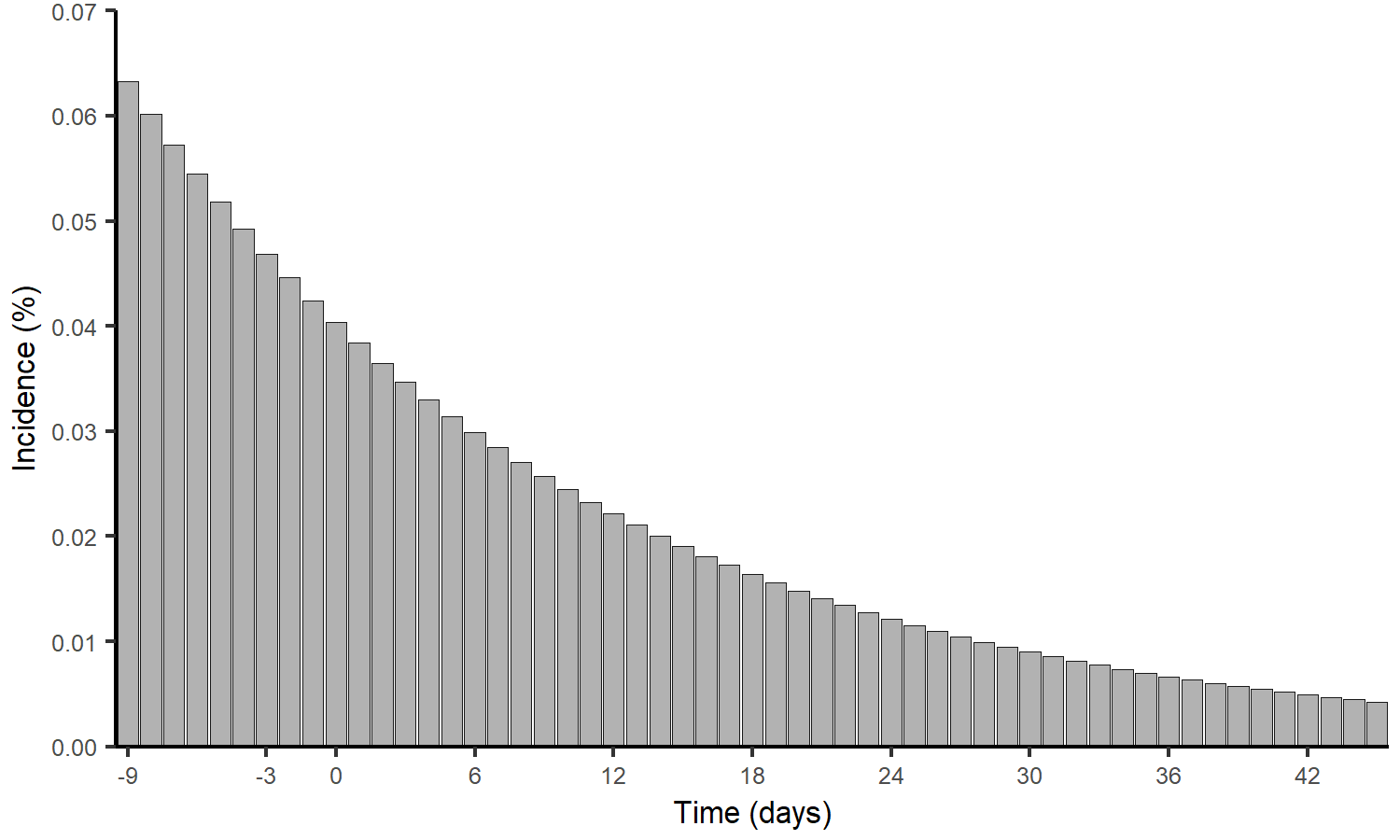
**

**Supplementary Figure 4.** Simulated daily incidence as a percentage of the entire population matching parameter assumptions in main text Figure 1.

**Supplementary Materials 1: Methods**

We used a simple discrete time model to simulate infection and test states in a population over a short period of exponentially growing or declining infection incidence. The model generates infections, prevalence, overall LFT and qPCR test positivity, and isolation status of detected individuals under a strategy of repeated LFT testing. In the main text, model parameters were chosen to reflect exponentially declining incidence with prevalence and population size set to represent the number of screened secondary school aged children in England. We describe the three key components of the model:

1. The incidence and prevalence of infections.
2. Characteristics of test sensitivity and specificity.
3. Testing strategy and subsequent isolation.

**Incidence of new infections**

We assume that new infections, $I(t)$, arise each day under an exponential growth curve with a specified growth rate and cumulative incidence:

$$I(t) =N\psi\frac{e^{\beta t}}{\sum_{i=1}^{t_{max}} e^{\beta i}}$$

Where $N$is the population size (set to 1.5 million); $t_{max}$ is the duration of the simulation period (set to 97 days, with the first 42 days discarded as burn-in; note that the simulation runs for 55 days but the 3-day LFT screening strategy only begins 9 days after burn-in); $\beta$ is the exponential growth rate (set to -0.05); and $\psi$ is the cumulative incidence between $t=1$ and $t=t_{max}$ (set to 0.1). We assume that each infected individual remains truly positive (i.e., contributes towards true prevalence) for $\gamma$ days, set to 21 days in the main text.

**Test characteristics**

Each infected individual is tested daily with qPCR and LFT to find the number of tests returning positive under daily, complete cross-sectional surveillance. For infected individuals, each day post infection, the result of a test is simulated as a Bernoulli trial with probability of returning a true positive equal to $P_{LFT}(x)$ or $P_{qPCR}(x)$, representing the probability of returning a positive LFT or qPCR result conditional on having been infected $x$ days prior. $P_{LFT}(x)$ and $P_{qPCR}(x)$ were defined by scaled gamma distributions:

$$f(x)=\frac{1}{\Gamma(k)\theta^{k}}x^{k-1}e^{\frac{-x}{\theta}}$$

$$P_{LFT/qPCR}(x)=min(\frac{f(x)}{\lambda*max(f(x))},p_{max})$$

Where $f(x)$ is the probability density function of the gamma distribution with shape parameter $k$ and scale parameter $\theta$; $\lambda$ is an arbitrary scaling parameter that sets the maximum value for $f(x)$ at the mode; and $p_{max}$ is the maximum sensitivity. Note that $\lambda$ can take values above 1 to generate a plateau of maximally sensitive test days, truncated by $p_{max}$.

For uninfected individuals, false positive results are simulated from Bernoulli trials with probability equal to one minus the specificity, $1-sp_{LFT}$ or $1-sp_{qPCR}$. For the main text results, we assumed that $sp_{LFT}=99.97\%$ and $sp_{qPCR}=99.99\%$.

The gamma distribution was chosen to generate a flexible parametric curve representing how test sensitivity increases then decreases over the course of an infection [[1]](https://paperpile.com/c/p0qVv1/YuWe). If the LFT is taken around the time of peak viral load then sensitivity will be high, whereas if the sample is taken three weeks after infection onset then sensitivity will be far lower [[2]](https://paperpile.com/c/p0qVv1/53Wp). qPCR sensitivity demonstrates the same time dependence as LFTs but with higher sensitivity early and late in the infection due to its very low analytical limit of detection. qPCR also returns a positive test result for individuals who are no longer infectious but may still shed viral RNA for a long time after recovery.

**Surveillance strategies**

As a benchmark, we simulated test results assuming that all individuals are tested each day of the simulation with qPCR or LFT, with no isolation of detected positives. We compared this to the results of testing all individuals with an LFT every $\rho$ days, where $\rho=3$ in the main text. In this strategy, individuals who are detected as positive enter isolation for 10 days following detection and cannot be tested again. True positives in isolation are categorised as “correctly isolating”, whereas false positives in isolation are categorised as “incorrectly isolating”. True infections who are not detected at any point in their infection are categorised as “not detected”.

**Undetected infections**

Under the simulation, there were a total of 150,000 infections. The vast majority of these occurred in the discarded burn-in phase of the simulation: 7050 infections arose in the nine days prior to initiation of routine screening, and 11,200 thereafter. 190 of the infections from the nine days prior to routine screening and 270 infections thereafter were never detected. However, 250/270 of these latter infections occurred in the final week of the simulation and may have been captured if routine screening continued. Only 20 of the 10600 infections which arose within the screening period and more than one week prior to the end of the simulation were missed.

**Supplementary references**

1. [Smith RL, Gibson LL, Martinez PP, Ke R, Mirza A, Conte M, et al. Longitudinal assessment of diagnostic test performance over the course of acute SARS-CoV-2 infection. medRxiv; 2021. doi:](http://paperpile.com/b/p0qVv1/YuWe)[10.1101/2021.03.19.21253964](http://dx.doi.org/10.1101/2021.03.19.21253964)

2. [Kucirka LM, Lauer SA, Laeyendecker O, Boon D, Lessler J. Variation in False-Negative Rate of Reverse Transcriptase Polymerase Chain Reaction-Based SARS-CoV-2 Tests by Time Since Exposure. Ann Intern Med. 2020;173: 262–267. doi:](http://paperpile.com/b/p0qVv1/53Wp)[10.7326/M20-1495](http://dx.doi.org/10.7326/M20-1495)
